# Interactions in the bond-frustrated helimagnet ZnCr_2_Se_4_ investigated by NMR

**DOI:** 10.1038/s41598-019-52962-4

**Published:** 2019-11-12

**Authors:** Sejun Park, Sangil Kwon, Soonchil Lee, Seunghyun Khim, Dilip Bhoi, Chang Bae Park, Kee Hoon Kim

**Affiliations:** 1 KAIST, Department of Physics, Daejeon, 34141 Republic of Korea; 2 University of Waterloo, Institute for Quantum Computing and Department of Physics and Astronomy, Waterloo, Ontario N2L 3G1 Canada; 3 Seoul National University, Center for Novel States of Complex Materials Research, Department of Physics and Astronomy, Seoul, 08826 Republic of Korea; 4 Seoul National University, Institute of Applied Physics, Department of Physics and Astronomy, Seoul, 08826 Republic of Korea

**Keywords:** Magnetic properties and materials, Molecular electronics

## Abstract

The zero field ^53^Cr nuclear magnetic resonance was measured at low temperatures to investigate the interactions in the bond-frustrated *S* = 3/2 Heisenberg helimagnet ZnCr_2_Se_4_. A quadratic decrease of the sublattice magnetization was determined from the temperature dependence of the isotropic hyperfine field. We calculated the magnetization using linear spin wave theory for the incommensurate spiral spin order and compared this outcome with experimental results to estimate the coupling constants. The hyperfine fields at Cr and Se ions provide evidences that the spin polarization of Cr ions is transferred to neighboring Se ions due to the covalent bonding between them, resulting in reduced magnetic moment in the Cr ion. This observation indicates that the Jahn-Teller effect, which leads to distortion inducing spin-lattice coupling, is not completely missing in ZnCr_2_Se_4_.

## Introduction

In spinels consisting of a chromium and nonmagnetic ions, ACr_2_X_4_ (A = Zn, Cd, Hg and X = O, S, Se), the combination of A and X ions allows control over the distance between neighboring Cr ions resulting in a broad range of interaction strength. The antiferromagnetic direct exchange interaction is dominant in ZnCr_2_O_4_ given the narrow distances between Cr ions. The tetrahedral structure of Cr ions subsequently predicts the three dimensional geometrical frustration^1,2^. At greater distances between the Cr ions, as in HgCr_2_Se_4_, the ferromagnetic superexchange interaction is dominant over the direct exchange interaction^3–5^. In ZnCr_2_Se_4_, the Cr–Cr distance is between these two. The resulting interaction is ferromagnetic as manifested by a positive Curie-Weiss temperature of *θ*_*cw*_ = 90 K^6,7^, but the actual spin order occurring at T_*N*_ = 21 K^8^ is incommensurate helical. This discrepancy implies that nearest-neighbor interaction is comparable and opposite to farther neighbor interactions, that is, bond frustration plays an important role.

ZnCr_2_Se_4_ undergoes a structural transition from cubic to tetragonal (c/a = 0.9992)^9^ concurrent with the magnetic phase transition. It also exhibits negative thermal expansion^8^ and high magnetostriction^10^. All of these observations provide evidence of strong spin-lattice coupling^11^. This is an interesting feature because three *d*^3^ electrons of a Cr^3+^ ion half-fill the *t*_2*g*_ triplet and the orbital angular momentum is quenched to zero. Therefore, the simultaneous transition of the structure and magnetic orders does not originate from the conventional Jahn-Teller (JT) distortion and magnetostriction effect is not due to spin-orbit coupling. The observed g-factor is in fact quite close to the spin-only value^8,12^. On the other hand, spin-lattice coupling in frustrated magnets can be generated by the spin driven JT effect^13^ that the frustration is released by structural distortion. That is, the strong spin-lattice coupling is the result of the competition between the ferromagnetic nearest neighbor interaction and the antiferromagnetic farther neighbor interactions.

**Figure 1:**
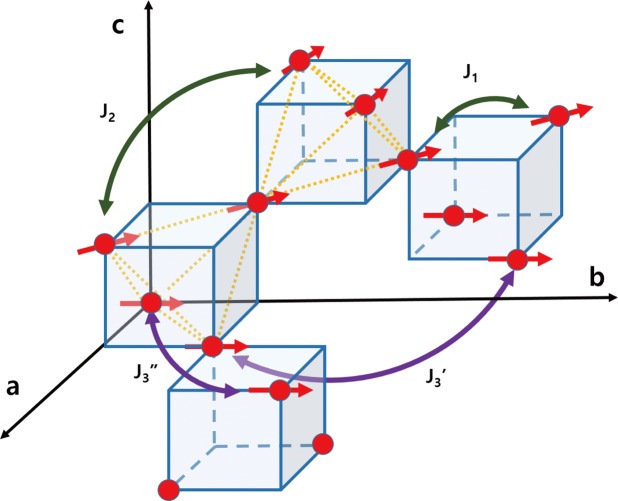
Crystal and spin structures of ZnCr_2_Se_4_. Red dots and arrows represent Cr ions and the directions of the ordered spins, respectively. Coupling constants *J*_*n*_ between the nth nearest neighbors are drawn up to the 3rd. Two different cases of *J*_3_ are marked as ${J}_{3}^{^{\prime} }$ and ${J}_{3}^{^{\prime\prime} }$. Some of the nearest neighbors are connected with yellow dotted lines.

Despite the necessity for information about these interactions to understand the spin-lattice coupling in ZnCr_2_Se_4_, reported values of the strength of the interactions have been inconsistent. The results of a neutron powder diffraction experiment provided suitable explanation with the combination of *J*_1_ ~ −1, *J*_2_ ~ 0.3 meV^14^, where *J*_*i*_ is the coupling constant between the *i*-th nearest Cr neighbors as shown in Fig. 1. However, a neutron scattering experiment with a single crystal reported *J*_1_ ~ −3, *J*_2_ ~ 0.03, *J*_3_ ~ 0.5, *J*_4_ ~ −0.04 meV^15^. A calculation of the local spin density approximation plus U also predicted that the magnitudes of ferromagnetic *J*_1_ and antiferromagnetic *J*_3_ are larger than those of *J*_2_ and *J*_4_ by one order, though estimated values^5^ were quantitatively different from those in the neutron scattering data.

The magnetic moment of Cr^3+^ ions has also been the subject of some debate. The fit of susceptibility data to the Curie-Weiss law produced 3 *μ*_*B*_ per Cr ion^12,16^. Contrary to macroscopic measurements, neutron diffraction studies have reported that the magnetic moment of a Cr^3+^ ion is not greater than 2 *μ*_*B*_^17,18^ and have suggested that spin fluctuation reduces the average magnetic moment. In contrast, a recent neutron diffraction study claimed that 3 *μ*_*B*_ was obtained in agreement with a spin-only Cr^3+^ ion and that smaller values may originate from the non-stoichiometry of samples^14^. In addition to spin fluctuation and non-stoichiometry, metal-ligand covalence can reduce the magnetic moment of a Cr ion in ZnCr_2_Se_4_. It is known that covalent hybridization spreads the spin polarization of a metal ion onto the ligand orbital^19^. Considering that covalence is relatively strong in selenides, this could in fact be the major cause of the reduced moment. The covalence is related to the spin-lattice coupling either because the changed electron density in Cr ions would induce distortion.

In this paper, we report the coupling constants and the magnetic moment of Cr ions in ZnCr_2_Se_4_ as obtained by zero-field nuclear magnetic resonance (NMR). Temperature dependence of the magnetic moment *M*(*T*) was obtained from the isotropic part of the hyperfine field. The sublattice magnetization was calculated by applying linear spin wave theory to the incommensurate helical spin distribution, which was in good agreement with experimental outcomes. The exchange constants were estimated by fitting the theory to the experimental results. The evidences of strong Cr-Se covalence were provided by the isotropic and anisotropic hyperfine fields obtained by Cr and Se NMR.

## Results

**Figure 2:**
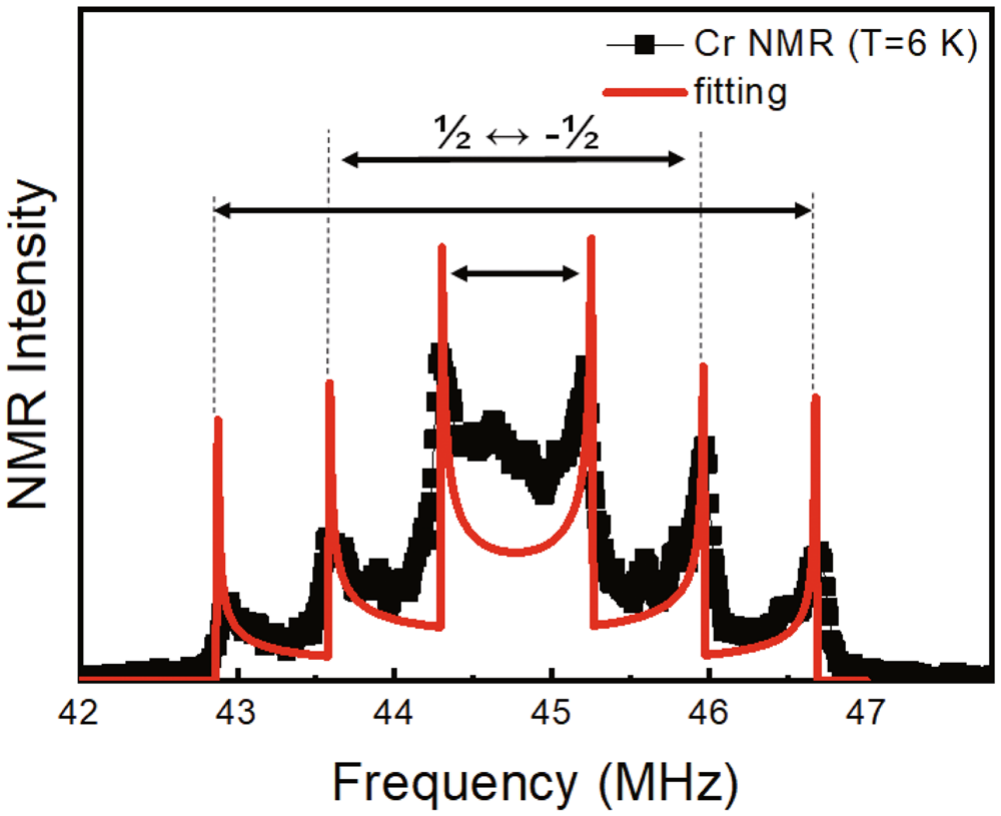
Zero-field NMR spectrum obtained at 6 K. Black points are experimental data and the red line is the fitting curve. The nuclear quadrupole resonances between the 3/2 ↔ 1/2, 1/2 ↔ −1/2, and −1/2 ↔ −3/2 levels are depicted by the arrow.

The zero-field ^53^Cr NMR spectrum obtained from polycrystalline ZnCr_2_Se_4_ at 6 K is shown in Fig. 2. As temperature increases to 15 K, the center frequency decreases and the peak positions shift a little while the spectral shape consisting of six peaks remains the same. This spectral shape is due to the anisotropic hyperfine and nuclear quadrupole interactions both of which produce U-shaped spectra when diverse spin directions generate diverse internal magnetic fields. In materials with an incommensurate helical spin structure such as BiFeO_3_, the spins pointing in all directions produce such a spectrum^20,21^. A similar spectrum is observed when an external magnetic field generates diverse spin directions in powder samples^22^. Because spectrum was obtained in a zero external field, the observed U-shape is not the characteristics of powder samples but instead represents the incommensurate spin helical structure of ZnCr_2_Se_4_.

The NMR resonance frequency for magnetic materials is determined by the interaction

1 $$H={H}_{iso}+{H}_{ani}+{H}_{NQR},$$

where H_*iso*_, H_*ani*_, and H_*NQR*_ represent the isotropic and anisotropic hyperfine interactions, and the nuclear quadrupole interaction, respectively. The ordered electron spins in the *d* orbital polarize the spins in the inner *s*-shell, generating a strong isotropic hyperfine field at the nuclei through Fermi contact interaction. The spins in the same and neighboring atoms generate dipolar hyperfine fields which are anisotropic. The spectral shape expected from these interactions for an incommensurate helical spin distribution is derived in the Method.

**Figure 3:**
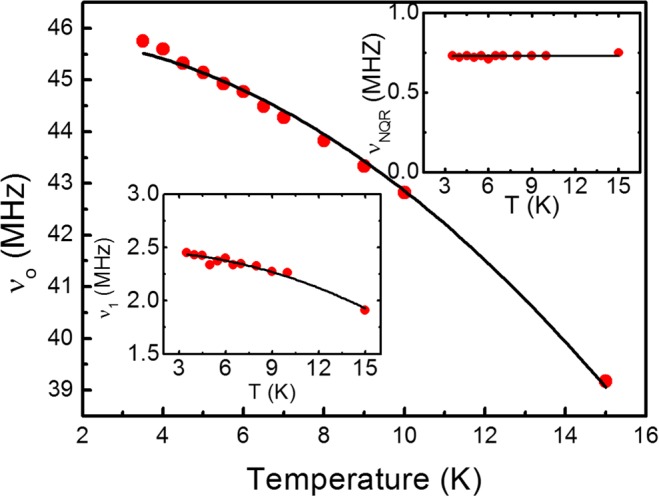
NMR frequency of the isotropic hyperfine field, *ν*_0_, versus the temperature. The black solid line is the *T*^2^ fitting curve. NMR frequency decreases in proportion to the square of the temperature. The lower left inset is the NMR frequency of the anisotropic hyperfine field, *ν*_1_, and the upper right inset is the NQR frequency, *ν*_*NQR*_ in the same temperature range.

We fit the data in Fig. 2 using Eq. (9) in Method. The fitting parameters are *ν*_0_, *ν*_1_, and *ν*_*NQR*_, which are the resonance frequencies of the isotropic and anisotropic hyperfine fields, and the nuclear quadrupole resonance (NQR) frequency, respectively. The result of the fit drawn together in the Fig. appears to be quite satisfactory. The temperature dependence of *ν*_0_, *ν*_1_, and *ν*_*NQR*_ obtained from the fit to the NMR spectra measured at various temperatures are shown in Fig. 3 and the insets therein. *ν*_0_ shows a monotonic decrease of approximately 15% as the temperature increases from 4 K to 15 K. *ν*_1_ drawn in the lower left inset, also decreases by a similar amount. Because hyperfine fields are proportional to the magnetic moment of the electron spins, *ν*_0_ and *ν*_1_ are proportional to the electron magnetic moment either. Therefore, they should change with the temperature in the same way. The NQR frequency depends on the ion position in a crystal structure and is therefore independent of the temperature. *ν*_*NQR*_ drawn in the upper right inset remains constant with the temperature, as expected. The same temperature dependence of *ν*_0_ and *ν*_1_ and the temperature-independent *ν*_*NQR*_ strongly supports the legitimacy of the analysis.

## Discussion

Since *ν*_0_ is proportional to the electron magnetic moment as mentioned above, the main graph in Fig. 3 represents the temperature dependence of sublattice magnetization. To determine the experimental coupling constant from our data, we calculated the temperature dependence of the magnetic moment in a helical order using linear spin wave theory. The spin Hamiltonian

2 $$H=\sum _{i,j}\,{J}_{ij}{\overrightarrow{S}}_{i}\cdot {\overrightarrow{S}}_{j}$$

can be diagonalized by the lowest order of the Holstein-Primakoff transformation followed by the Bogoliubov transformation in the form of $H={\sum }_{k}\,\hslash {\omega }_{k}{\alpha }_{k}^{\dagger }{\alpha }_{k}$ with the dispersion relation^15^ of

3 $${\omega }_{k}=\frac{S\hslash }{4}\sqrt{({J}_{k}-{J}_{Q})(\frac{1}{2}{J}_{k+Q}+\frac{1}{2}{J}_{k-Q}-{J}_{Q})}$$

for a helical spin order with wave vector *Q*, where *J*_*k*_ is the Fourier transform of *J*_*ij*_.

The temperature dependence of the magnetic moment <*S*^*z*^> is obtained from the Bose-Einstein distribution of magnons as

4 $$\frac{ < {S}^{z} > }{S}\,=\,1-\sum _{k}(\frac{{X}_{k}+{Y}_{k}}{4\sqrt{{X}_{k}{Y}_{k}}}-\frac{1}{2})-\sum _{k}\frac{1}{{e}^{\frac{\hslash {\omega }_{k}}{{k}_{B}T}}-1}\frac{{X}_{k}+{Y}_{k}}{2\sqrt{{X}_{k}{Y}_{k}}},$$

where *X*_*k*_ ≡ *J*_*k*_ − *J*_*Q*_ and ${Y}_{k}\equiv \frac{1}{2}{J}_{k+Q}+\frac{1}{2}{J}_{k-Q}-{J}_{Q}$. The second term is the magnetic moment reduction due to quantum fluctuation which is temperature-independent. The temperature dependence of magnetization comes from the third term. At low temperatures, only the low-energy spin wave excitations are considered. The dispersion relation in Eq. (3) shows that the main contribution to the summation over *k* comes mostly near |*k*| = |*Q*| and *k* = 0. However, it has been reported that magnon-magnon scattering opens gaps at (0, ±*Q*, 0) and (±*Q*, 0, 0)^15^. Therefore, we need to sum only around (0, 0, 0) and the Goldstone modes centered at (0, 0, ±*Q*). Then it can be shown that the magnetic moment reduction depends on temperature as

5 $$\Delta S=\alpha ({J}_{ij})({k}_{B}T{)}^{2},$$

where *α*(*J*_*ij*_) is a constant depending on the coupling constants *J*_*ij*_. For example,

6 $$\begin{array}{rcl}\alpha ({J}_{ij}) & = & \frac{{\alpha }_{0}}{\mathrm{(1}+cos\gamma ){J}_{1}+\mathrm{8(1}+cos2\gamma ){J}_{3}}\frac{1}{cos\gamma {J}_{1}+8cos2\gamma {J}_{3}}\\  &  & \times \,(\frac{1}{\sqrt{\mathrm{(1}-cos\gamma ){J}_{1}+\mathrm{2(1}-cos2\gamma ){J}_{3}}}\\  &  & +\,\frac{2}{\sqrt{(\frac{1}{2}-cos\gamma +\frac{1}{2}cos2\gamma ){J}_{1}+\mathrm{(1}-2cos2\gamma +cos4\gamma ){J}_{3}}})\end{array}$$

if the Hamiltonian in Eq. (1) contains only two coupling constants, *J*_1_ and *J*_3_. Here *γ* is the angle between the spins in the neighboring *ab* planes. The magnetic moment decreases in proportion to T^2^, as in antiferromagnets.

**Figure 4:**
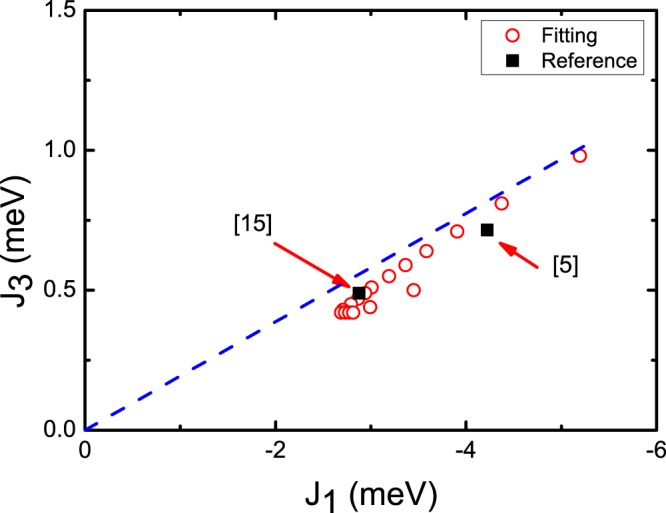
*J*_1_ − *J*_3_ pairs obtained from the fitting of Fig. 3 (red open circles). The dashed line represents the relationship *J*_3_ = −0.2 *J*_1_ (see the main text). Black squares are previously reported theoretical^5^ and experimental^15^ values.

The fit of the data in Fig. 3 to *αT*^2^ gives *α* = 33(±1) × 10^−3^ MHz/K^2^. The result is depicted as the black solid curve in the figure. It appears that the theoretical model describes the data well. Considering the uncertainty in the estimation of *α* by the fit, we searched for *J*_1_ and *J*_3_ pairs which gives |*α*(*J*_*ij*_) − *α*|/*α* < 0.03. All the pairs of *J*_1_ and *J*_3_ satisfying this condition are depicted as red open circles in Fig. 4. There are two different couplings between the third-nearest neighbors (${J}_{3}^{^{\prime} }$ and ${J}_{3}^{^{\prime\prime} }$, see Fig. 1) but we simply used the same *J*_3_ value because the difference is negligible^5^.

As |*J*_1_| increases, the points approach asymptotically a linear curve that passes through the origin, ${J}_{3}\, \sim \,-\,0.2\,{J}_{1}$. This means that the same helical order can be generated for different pairs of ferromagnetic *J*_1_ and antiferromagnetic *J*_3_ as long as their ratio is fixed. When only *J*_1_ and *J*_3_ are considered, the ground-state energy of a helical order of classical spins is given by *E*(*γ*) = 4*J*_1_(*cosγ* − 1) + 8*J*_3_(*cos*2*γ* − 1)^5^. The ground-state configuration is obtained by requiring (*dE*/*dγ*)_*γ*__0_ = 0. The experimentally observed angle between the spins on the neighboring *ab* planes *γ*_0_ = 42° gives *J*_3_/*J*_1_ ~ −0.17, which roughly matches with the value estimated from the fit. The black squares in Fig. 4 represent *J*_1_ and *J*_3_ values reported in the experimental and theoretical works claiming that *J*_1_ and *J*_3_ are one order of magnitude stronger than *J*_2_ or *J*_4_^5,14,15^. Although these values are different from each other, they follow the relationship trend we introduce here.

We repeated the same fit procedure with *J*_1_ and *J*_2_ instead of *J*_1_ and *J*_3_. The result shows that the pairs of *J*_1_ > |−2.4| meV and *J*_2_ > 0.8 meV fit the data similarly. However, the result is incompatible with that of a neutron powder diffraction experiment which was explained with *J*_1_ ~ −1 and *J*_2_ ~ 0.3 meV^14^. The NMR result supports the bonding scheme with the ferromagnetic *J*_1_ competing with antiferromagnetic *J*_3_, rather than that with *J*_1_ and *J*_2_. We also attempted to fit the data to the form *T* ^3/2^*exp*(−*E*_*G*_/*k*_*B*_*T*) which is the temperature dependence of magnetization expected when energy gap *E*_*G*_ exists in the spin wave dispersion relation. The result was *E*_*G*_ = 1 ± 13 meV, indicative of practically no energy gap and, consistent with the neutron scattering experiment.

The NMR center frequency of the Cr^3+^ ions extrapolated to 0 K in Fig. 3 is about 46 MHz. Zero-field Cr NMR for other ACr_2_Se_4_ compounds was also observed at a similar frequency^23^. Cr NMR for ACr_2_O_4_ compounds is, however, observed in the frequency range of 68 ± 7 MHz^24–28^. The resonance frequency of Mn^2+^ NMR for manganese compounds is also reduced by about a third when O or F ion ligands are replaced by Se ions^27^. The difference in the resonance frequencies of the selenides and oxides is attributed to the difference in the magnetic moments and the hyperfine coupling constants. Given that the magnetic moment of Cr^3+^ ions in chromium oxide spinels is 3 *μ*_*B*_, the magnetic moment of the chromium selenide should be less than 3 *μ*_*B*_.

Supporting evidences for the reduced magnetic moment of Cr^3+^ ions are also provided by the Se NMR and the anisotropic hyperfine field at a Cr ion. When macroscopic measurements of the magnetic moment gives 3 *μ*_*B*_ per Cr ion^12,16^, and one Cr ion has less than 3 *μ*_*B*_, the remaining moment should be found in the other ions of ZnCr_2_Se_4_. In general, the covalence increases in metal-ligand bonding as the oxygen ligand is replaced by sulfur, and sulfur by selenium. Covalent hybridization due to the strong overlap between the Cr 3d and Se 4p orbitals is expected to transfer the spin polarization of Cr ions to Se ions^27^. We measured the transferred hyperfine field at the Se nuclei of ZnCr_2_Se_4_ by zero-field Se NMR. The resulting hyperfine field strength is 8 ~ 10T, which is about half of that in Cr nuclei, 18.3 T. Cr and Se NMR for CdCr_2_Se_4_ and HgCr_2_Se_4_ show the similar results^23^. This large hyperfine field in nonmagnetic Se ions provides one clear evidence of a large spin polarization transfer from Cr ions. It is known that the intensity of spin fluctuations measured in neutron scattering experiment decreases due to reduced magnetic moment associated with such strong covalence^19,29^.

The anisotropic hyperfine field is generated by *L* or *S* at the same or neighboring atoms. In the simple ionic picture in which three *d* electrons occupy *t*_2*g*_ orbitals, the orbital angular momentum *L* is quenched and the hyperfine field due to *S* at the same atom is isotropic because the shape of the half-filled *t*_2*g*_ orbitals is spherical. Therefore, only the dipolar field due to *S* in the neighboring Cr ions can contribute to the anisotropic hyperfine field. We calculated the dipolar field generated by the helically ordered spins in ZnCr_2_Se_4_. Considering up to the third nearest neighbors, the sum of the dipolar field is estimated to be approximately 340 mT. Extrapolation of the anisotropic hyperfine field data in the inset of Fig. 3 to 0 K gives a value of 2.5 MHz, which approximately corresponds to 1T.

Because the dipolar field is only a third of the observed value, the major anisotropic hyperfine field should be generated in the same atom. This means that either there should be an unquenched *L* or that the orbital is not exactly spherical. The g-factor measured along the [111] direction of a single crystal^8^ is identical to that obtained from poly-crystals^12^, 1.996. Therefore, the unquenched orbital angular momentum is negligible and the most plausible cause of the anisotropic hyperfine field is the spin in the non-spherical *d* orbital. The orbital in Cr^3+^ ions might become non-spherical when the effective number of electrons changes from three by covalence. A small fraction of a pair of up and down electrons is shifted from Se to Cr ions in the covalent bonding, and a similar fraction of one electron is shifted in the opposite direction in the antibonding^30^. In this way, a net electron is transferred from a Se ion to a Cr ion, while a spin polarization is transferred in the opposite direction. The changed electron density in Cr ions should then induce distortion together with the spin-driven JT effect, generating spin-lattice coupling in ZnCr_2_Se_4_ where the JT effect is missing from the pure ionic point of view.

In summary, the strong spin-lattice coupling in ZnCr_2_Se_4_, where the conventional JT effect is missing, can be explained by the spin driven JT effect. That is, the spin-lattice coupling in the frustrated magnet ZnCr_2_Se_4_ is the result of competing ferromagnetic and antiferromagnetic couplings. To determine the coupling constants, we obtained *M*(*T*) from the temperature dependence of the zero-field Cr NMR spectrum and compared with theory. We derived $M(T) \sim {T}^{2}$ using linear spin wave theory. The comparison supports the claim that *J*_1_ and *J*_3_ are dominant couplings rather than *J*_1_ and *J*_2_. The result yielded the asymptotic relationship *J*_3_ ~ −0.2*J*_1_ for large couplings.

We have shown evidences for the spin polarization transfer from Cr ions to Se ions due to strong covalence. First, the strength of the isotropic hyperfine field implies that the effective number of *d* electron spins in a Cr ion is less than three. Second, the anisotropic hyperfine field indicates that the total *d* electron orbital is not spherical. Third, Se NMR supports a large spin polarization transfer from Cr ions to Se ions. The changed number of electrons can then induce distortion of the lattice in addition to the spin-induced JT distortion. Covalence plays a role in the spin-lattice coupling in ZnCr_2_Se_4_.

## Methods

### NMR experiment

For NMR experiments, polycrystalline ZnCr_2_Se_4_ samples were used because single crystals produced much lower signal intensity levels, most likely due to conductivity. The samples were prepared in a closed quartz ampule using a stoichiometric quantities of Zn (99.99%), Cr (99.999%), and Se (99.999%). The elements were thoroughly mixed, pressed into pellets, and heated to 650 °C for 24 hrs. The product was reground and the same heating procedure was repeated once more. The phase purity of the polycrystalline sample was checked at room temperature via powder X-ray diffraction. All experiments were performed without external magnetic fields. A conventional 90-*τ*-180 pulse sequence was used to obtain the spin echo signal at temperatures lower than T_*N*_. The echo signal was measured in a wide frequency range between 35 and 50 MHz at an interval of 0.1 MHz with rf pulses of 0.25 MHz bandwidth. The full NMR spectrum was obtained by summing the Fourier-transformed signals^31^.

### NMR spectrum analysis

Considering all three interactions in Eq. (1), the NMR resonance frequency is given as follows:

7 $$\nu ={\nu }_{{\rm{iso}}}+{\nu }_{{\rm{ani}}}(3{co}{{s}}^{2}\alpha -1)+{\nu }_{{\rm{NQR}}}(3{co}{{s}}^{2}\beta -1).$$

Here, *α* and *β* represent the angles between the spin direction and the anisotropy axis of the hyperfine field and the principal axis of the NQR, respectively. *ν*_0_ and *ν*_1_ are the resonance frequencies of the isotropic and anisotropic hyperfine fields, respectively, and the first order NQR frequency for *m* → *m* − 1 transition, *ν*_*NQR*_ = −(3*e*^2^*qQ*/2*hI*(2*I* − 1))(*m* − 1/2)/2. Because the spins of ZnCr_2_Se_4_ lie on the ab plane, the spin direction is describable with only the azimuthal angle *ϕ*. The crystal symmetry implies that the NQR principal axis is along the [111] axis, along which Cr ions are located symmetrically. It is known CdCr_2_Se_4_ has the principal axis of NQR in this direction^32^. The angle dependence of the NQR term, 3cos^2^*β* − 1, can then be replaced by sin(2*ϕ*). Expressing the angle dependence of the anisotropic hyperfine field in terms of *ϕ*, Eq. (7) can be rewritten as^20,21^

8 $$\nu ={\nu }_{0}+{\nu }_{1}{si}{{n}}^{2}(\varphi -\delta )+{\nu }_{{\rm{NQR}}}\,\sin (2\varphi ),$$

where *δ* is the azimuthal angle of the axis of the anisotropic hyperfine field. *ν*_0_ is the sum of all angle-independent terms and *ν*_1_ is the value of *ν*_*ani*_ projected on the ab plane.

A simple trigonometric calculation of Eq. (8) gives

9 $$\nu =({\nu }_{0}+\frac{{\nu }_{1}}{2})+\sqrt{{{\nu }_{{\rm{NQR}}}}^{2}+\frac{{\nu }_{1}^{2}}{4}-{\nu }_{1}{\nu }_{{\rm{NQR}}}\,\sin (2\delta )}\,\sin (2\varphi -{\varphi }_{0}).$$

Here, *ϕ*_0_ is a constant. The equation shows that the frequency change with the azimuthal angle is minimal at every 90 degrees. In an incommensurate spiral spin distribution, all spins are uniformly distributed in the angle domain. Such a spatially uniform distribution appears as a U-shaped distribution in the frequency domain. NQR gives three different spectral shifts *m* = 3/2, 1/2, −1/2, to split the spectrum into three. As a result, the superposition of three U-shaped spectra with six peaks is generated.

We fit the data in Fig. 2 using Eq. (9). The center frequency *ν*_0_ + *ν*_1_/2 was determined according to the symmetry point of the spectrum, and the fitting parameters were *ν*_1_, *ν*_*NQR*_, and *δ*. The result of the fit drawn together in Fig. 2 appears to be quite satisfactory. The best fit resulted in *δ* = *π*/4, meaning that the axis of the anisotropic hyperfine field is on the (1$\bar{1}$0) plane. The principle axis of NQR is along the symmetry axis of Cr ions, i.e., the [111] direction. Because this axis is on the (1$\bar{1}$0) plane, the fitting result strongly implies that the axis of the anisotropic hyperfine field coincides with the principle axis of the NQR. The NMR signal of CdCr_2_Se_4_, having a crystal structure identical to that of ZnCr_2_Se_4_, was also suitably described using the same axis of the anisotropic hyperfine field^32^.

## Data Availability

The datasets generated during and/or analysed during the current study are available from the corresponding author on reasonable request.
